# Determinants of preterm birth among newborns delivered at public referral hospitals in Bahir Dar City, Northwest Ethiopia, 2024

**DOI:** 10.1186/s12889-025-24024-0

**Published:** 2025-08-25

**Authors:** Ayalew Kassie, Muluken Assefa, Wubante Demilew, Gebeyaw Derso, Tadesse Ayana Fentie, Amanuel Taye, Melese Alemnew Ayal, Bazezew Asfaw Guadie, Zewudu Bishaw Aynalem, Mulugeta Kassie, Berihun Bantie, Gebrie Getu Alemu

**Affiliations:** 1 Department of Nursing, Amhara Regional Health Bureau, Bahir Dar Health Science College, P. O. Box 684, Bahir Dar, Ethiopia; 2 Department of Nursing, College of Health Sciences, Injibara University, P.O. Box 40, Injibara, Ethiopia; 3 Department of Nursing, Amhara Regional Health Bureau, Wogeda Primary Hospital, Bahir-Dar, Ethiopia; 4 Department of Nursing, College of Medicine and Health Sciences, Debre Tabor University, P.O. Box 272, Debre Tabor, Ethiopia; 5 Department of Epidemiology and Biostatistics, Institute of Public Health, College of Medicine and Health Sciences, University of Gondar, Gondar, Ethiopia

**Keywords:** Determinants, Preterm birth, New born, Ethiopia

## Abstract

**Background:**

Preterm birth, defined as any birth occurring before 37 completed weeks of gestation, remains a significant global health concern and is a leading cause of neonatal morbidity and mortality in Ethiopia. While previous studies have highlighted maternal age, antenatal care attendance, and birth spacing as major determinants, this study focuses on exploring less examined factors specifically maternal behavioral patterns and nutritional status among mothers delivering at Bahir Dar Public Referral Hospitals.

**Objective:**

This study aimed to identify the determinants of preterm birth among newborns delivered at public referral hospitals in Bahir Dar City, Northwest Ethiopia, in 2024.

**Methods:**

Institution-based unmatched case-control study was conducted using a sample of 321 participants at public referral hospitals in Bahir Dar City from September 1st to December 30th, 2024. Newborns delivered before 37 weeks of gestation were cases and two newborns delivered at 37 and above weeks at the time that each case occurred were controls by using a systematic sampling technique. Data were analyzed using both bivariable and multivariable binary logistic regression models. All variables with a *p*-value of less than 0.20 in the bivariable analysis were included in the multivariable regression; statistical significance was set at *p* < 0.05.

**Results:**

A total of 107 cases and 214 controls participated with a response rate of 100%. Five determinants of preterm birth were found to be major ones. Newborns from urban residence (AOR: 0.21, 95% CI: 0.07, 0.60, *p*-value, 0.003) was a protective factors of preterm birth. Conversely, a large family size size six or more (AOR: 29, 95% CI: 6.29, 140.51, *p*-value, < 0 0.001), women with a birth interval of less than two years between consecutive births (AOR: 17, 95% CI: 4.77, 65.91, *p*-value, < 0.001), women who had no antenatal care (ANC) follow-up (AOR: 5, 95% CI: 1.68, 14.77, *p*-value, 0.004), and women who did not take iron with folic acid during pregnancy (AOR: 5, 95% CI: 1.69, 18.29, *p*-value, 0.005) were risk factors associated with preterm births.

**Conclusion and recommendations:**

The protective factor was urban residence while the number of family members 6 and above, less than 2 years of the intervals between births, lack of ANC follow-up, and lack of iron with folic acid supplementation were significant risk factors with preterm birth. The regional health bureau should reduce the gap between urban and rural health disparities, increase ANC coverage, reduce determinant risk factors, and enhance protective factors of preterm birth to improve maternal and neonatal health outcomes.

## Introduction

Preterm birth(PTB), defined as birth before 37 weeks of gestation from the first day of a woman’s last normal menstrual period (LNMP) [1]. PTB is categorized into moderate preterm (32-36 weeks), very preterm (28 −31 weeks), and extremely preterm (< 28 weeks) ADDIN EN.CITE [2–4]. The risk of neonatal morbidity and mortality increases as gestational age decreases ADDIN EN.CITE [2–4]. Infections are primarily associated with extremely preterm births, while stress and lifestyle factors are more commonly linked to moderate preterm births. Very preterm births often result from a combination of both infections and behavioral factors [4].

Globally, an estimated 15 million preterm birth occur each year, with 81% of these in Sub-Sahara Africa and South Asia [5]. PTB rates are highest in Africa (11.9%) and lowest in Europe (6.2%) [6]. The majority of global preterm births occur in Asia and Africa, where health systems are weak and access to healthcare services is limited [7]. Preterm birth is the leading cause of neonatal deaths, accounting for 18% of child deaths and 35% of deaths in newborns [8]. In 2015, approximately 500, 000 neonatal deaths due to preterm birth occurred in Sub-Saharan Africa [8]. Despite advances in prenatal and neonatal care, preterm infants face higher risks of developmental disabilities and health issues.

In Ethiopia, PTB remains a significant challenge. Each year, around 320,000 babies are born preterm, contributing to 24,000 deaths in children under five due to complications of PTB [9]. According to various studies in Ethiopia, the prevalence rate of preterm birth was 25.9% in Jimma [10], and 16.9% in Shire [11]. The national prevalence of preterm birth and neonatal death are 10.48% and 29.31% respectively ADDIN EN.CITE [9, 12–14] with considerable regional variations ranging from 12.8% in the Central Zone of Tigray [15] to 16.5% in Addis Ababa [16].

In the Amhara Region, the prevalence rate of preterm birth 14.3% [17]. Still, the burden of PTB remains high in the Amhara National Regional State (ANRS), where 1 in 8 (12.63%) PTB die during neonatal period [18]. It is the leading cause of prenatal morbidity and mortality in Ethiopia including in the region Amhara [19] and the second leading cause of under-5 mortality, after pneumonia [20].

Despite efforts, Ethiopia has not seen a significant reduction in PTB and neonatal mortality rates. In the majority of cases, preterm deliveries occur spontaneously and are associated with long-term complications among surviving infants [21].

WHO’s 2015 recommendations on possible interventions could save nearly 300,000 preterm infants in Sub-Saharan Africa through prioritized implementation and prevention strategies [22]. The year 2016 marked the beginning of the implementation of the Sustainable Development Goals (SDGs) [23]. The SDGs aim to reduce the Under-5 Mortality Rate (U5MR) to no more than 25 per 1, 000 live births in every country by 2030 [24]. Ethiopia also has signed this agreement aimed to reduce neonatal mortality to at least 12 per 1, 000 live births and under-5 mortality to at least 25 per 1,000 live births by 2030 under SDG 3.2 [8].

To effectively plan for elimination of preventable child deaths, it is essential to understand the current distribution of causes of child mortality, which has evolved over recent decades. Preterm birth leads to significant health consequences for the infant and imposes economic costs on families and communities. Advances in prenatal and neonatal care have enhanced the survival rates of preterm infants; however, those who survive are at a higher risk of developmental disabilities, health issues, and growth problems compared to infants born at full term [25].

This study identifies key determinants of preterm birth, aiming to support the achievement of the SDG child health targets by highlighting factors that can reduce preventable child mortality. It also contribute to progress towards SDG 3.2 in the region by informing the design of evidence-based interventions. This study aimed to identify the determinants of preterm birth among newborns delivered at public referral hospitals in Bahir Dar city, Northwest Ethiopia, in 2024.

The findings will be particularly valuable for clinicians, communities, programmers’ planners, and researchers:

- For clinicians: It helps prioritize counseling for high-risk mothers during ANC visits, strengthens preventive interventions, and promotes regular ANC attendance, iron-folic acid supplementation, and birth spacing education.
- For the Community: The study highlights the importance of ANC follow-up and birth spacing, promoting awareness and behavioral change to empower families to seek maternal care. It also strengthens community trust in health services.
- For Health Programmers: The identified risk factors provide evidence for designing targeted maternal and child health programs, especially in rural areas and among high-parity households, including scaling up ANC coverage, iron-folic acid availability, and family planning.
- For Researchers: This study contributes to understanding preterm birth determinants in low-resource settings, offering a foundation for further exploration of cultural and behavioral factors and developing intervention to reducing preterm birth rates.

## Objective

To identify the determinants of preterm birth among newborns delivered at public referral hospitals in Bahir Dar city, Northwest Ethiopia, 2024.

## Method and materials

### Study design and period

Intuitional based unmatched case control study was conducted from September 1^st^ to December 30^th^, 2024.

### Study area and population

This study was conducted at Felege Hiwot Comprhensive Referral Hospital (FHCSH) and Tibebe Ghion Comprhensive Specialized Referral Hospital (TGCSH), located in Bahir Dar City. Bahir Dar is a self-Administrative Zone in Northwest Ethiopia, situated 564 km away from Addis Ababa, the capital city of Ethiopia. Due to the range and quality of services offered, the city serves as a major healthcare destination for patients from various areas. According to the Bahir Dar city administration’s 2022 fiscal year report, the city’s population was estimated at 345,088 [26].

### Source population and study population

Women who had follow up and had referral to the selected hospitals are the source population.

### Study population

All women who gave birth in the selected referral hospitals during the study period were taken as study population.

### Sample size determination and sampling techniques

#### Sample size determination

Our study sample size was calculated based on the study conducted in Bahir Dar City Public Hospitals in 2019 which stated as determinant factors of preterm birth at public hospitals [27]. The sample size was calculated using 80% power, a 95% confidence interval, and the variable of birth spacing less than two years, which had an adjusted odd ratio (AOR) of 2.28. A case - to - control ratio of 1:3 and a proportion of 27.7 of controls among the exposed group were also considered. Accordingly, the initial calculated sample size was 291. After adding a 10% non-response rate, the final sample size became 321. The calculation was performed using EPi. Info Version 7.2.1.0.

#### Sampling techniques

Neonates with a gestational age of less than 37 weeks were defined as cases, and neonates with a gestational age of 37 weeks or more were defined as controls. The gestational age was established by the last normal menstrual period (LNMP) or an ultrasound examination. The controls and cases were recruited as soon as they were born. Two controls were recruited as soon as each case was happened.

The participants’ allocation was based on the average number of women who gave live birth at FHCSH and TGCSH, according to 2023 reported data. The total expected number of live births from 2023 to January 2024 was 5, 040 at FHCSH and 2, 484 at TGCSH. Based on the data of the two hospitals, FHCSH’s average monthly number of women who gave birth was 420, and TGCSH’s average number of women who gave birth was 207.

The number of study participants from each hospital was proportionally allocated. The number of expected live births at FHCSH was approximately twice that of TGCSH. Accordingly, out of the calculated sample size of 321, 214 participants allocated to FHCSH and 107 to TGCSH. For each identified case, data collectors subsequently interviewed two consecutive controls who met the inclusion criteria.

### Inclusion and exclusion criteria

#### Inclusion criteria

All immediate postnatal women who gave live birth and were willing to participate at the selected referral hospitals during the study period were included.

#### Exclusion criteria

Mothers who were unable to communicate during data collection, those with edema that could affect the accuracy of mid-upper arm circumference (MUAC) measurement, and those without a reliable Last Menstrual Period (LMP) or early ultrasound Scan (U/S) (i.e., performed at ≤20 completed weeks of gestation) were excluded from the study.

### Variables

#### Dependent variable

The outcome variable of the study was Preterm Birth.

#### Independent variables

The study examined several independent variables, including socio-demographic factors (maternal age, marital status, family size, education, residence, occupation, income, spouse’s education and occupation), and reproductive health variables (birth interval, antenatal care follow-up, number of ANC visits, gestational age at the start of ANC, and multiple pregnancies), and obstetric complications included antepartum hemorrhage (APH), premature rupture of membranes (PROM), history of preterm birth, stillbirth or abortion. Medical conditions like urinary tract infection (UTI), anemia, sexually transmitted infection (STI), hypertension, malaria, HIV/AIDS, and diabetes mellitus (DM) were also considered, along with behavioral and dietary factors such as consumption of home-brewed alcohol, mid-upper arm circumference (MUAC), and duration of iron supplementation.

### Operational definitions

Preterm birth occurs before 37 weeks (259 days) of gestation, measured from the first day of a woman’s last normal menstrual period or based on an ultrasound examination.

A previous birth defect is any birth outcome of a mother which were congenital malformed by history.

“Stillbirth is defined as a baby born with no signs of life at or after 28 weeks’ gestation” (WHO, 2006).

“Abortion is also defined as a fetus or embryo removed or expelled from the uterus during the first half of gestation (20 weeks or less), or born weighing < 500 g.“(National Center for Health Statistics, 1977).

Maternal Nutritional status: This specific WHO manual includes the classifications for malnutrition in emergency settings, including for pregnant women, and offers the MUAC cut-offs used in practices (World Health Organization, 1999). Sever Acute Malnutrition (SAM): MUAC < 18.5 cm; Moderate Acute Malnutrition (MAM): MUAC 18.5–21 cm; and Normal Nutritional Status: MUAC >21.0 cm.

### Data collection instrument

Data collection tool was adapted from the Ethiopia Demographic and Health Survey (EDHS) questionnaire [28].

The study focused on socio-demographic, obstetric, medical, and maternal nutritional factors. Preterm delivery was the outcome variable, while independent variables were obtained through direct interviews using a pretested questionnaire, following verbal consent.

Medical data were retrieved from patient records. Gestational age was determined by clinicians using the Last Normal Menstrual Period (LNMP) or Ultrasound (U/S). Mid-upper arm circumference (MUAC) was measured at mid-point of the left upper arm using flexible tape.

The questionnaire was English version and it was translated to local language. Finally, translated back-to English. Data were collected by two diploma nurses, who received a one-day orientation on the study’s purpose, respondent confidentiality, and their rights. A pretest was conducted at Addis Alem Primary Hospital, involving 10% (33 women) of the sample. The principal investigator and supervisors checked the data daily to ensure completeness and consistency.

### Data processing and analysis

Data were first checked for consistency and completeness before being entered into Open Epi Data version 3.1 and subsequently exported to STATA version 14 for analysis. Descriptive statistics were used to summarize participants’ characteristics. Bivariable and multivariable binary logistic regression analyses were conducted to identify factors associated with preterm birth. Variables with a *P*-value of less than 0.2 in the bivariable analysis were included in the multivariable model. In the final multivariable binary logistic regression model, statistical significance was determined at a *p*-value of less than 0.05. The strength of association was expressed using Adjusted Odds Ratio (AOR) with 95% confidence interval (CI).

## Results

### Socio-demographic related factors

Among all participants, 79.4% of the cases and 29.4% of the controls were from rural areas. Additionally, 57.9% of the women in the case group and 15.9% in the control group were uneducated. Regarding the educational status of spouses, 17.8% of the cases and 63.5% of the controls had attained a diploma or higher. In terms of family size, 93.5% of the women in the case group and 25.7% in the control group had a family size of six or more. Lastly, 49.5% of the cases and 38.3% the control groups had a family income below 5000 ETB (Ethiopian Birr) (Table 1).

**Table 1:** Socio-demographic characteristics of preterm birth among mothers who gave birth at public hospitals in Bahir dar, Amhara regional state, Northwest Ethiopia 2024 (*n* = 107 cases and 214 controls)

Predictor variables	Variable category	Cases	Controls	Statistics (X^2^), *P*-value
		N (%)	N (%)	
Residence	Rural	85(79.4)	63(29.4)	X^2^ = 74.94, *P* < 0.001
	Urban	22(20.6)	151(70.6)	
Age of mothers	18–25	38(35.5)	73(34.2)	X^2^ = 0.01, *P* > 0.05
	26–30	32(29.9)	69(32.2)	
	31–35	23(21.6)	51(23.9)	
	36–40	7(6.5)	9(4.1)	
	46 + years	7(6.5)	12(5.6)	
Marital status	Married	18(16.8)	185(86.5)	X^2^ = 142.71, *P* < 0.001
	Divorced	40(37.4)	18(8.4)	
	Widowed	49(45.8)	11(5.1)	
Age at first marriage	18–25	79(73.9)	200(93.5)	X^2^ = 6.05, *P* < 0.05
	26–30	13(12.1)	8(3.7)	
	Unknown	15(14.0)	6(2.8)	
Mothers’ level of education	Uneducated	62(57.9)	34(15.9)	X^2^ = 175.68, *P* < 0.001
	Secondary	39(36.5)	24(11.2)	
	Diploma and above	6(5.6)	156(72.9)	
Spouse education level	Uneducated	50(46.7)	21(9.9)	X^2^ = 75.37, *P* < 0.001
	Primary	25(23.4)	37(17.3)	
	Secondary	13(12.1)	20(9.3)	
	Diploma & above	19(17.8)	136(63.5)	
Mother’s work	House wife	42(39.3)	83(38.8)	X^2^ = 0.19, *P* > 0.05
	Civil servant	12(11.2)	28(13.1)	
	Merchant	31(29.0)	68(31.7)	
	Daily labor	22(20.5)	35(16.4)	
Spouse’s work	Farmer	17(15.9)	16(7.5)	X^2^ = 0.75, *P* > 0.05
	Merchant	46(43.0)	90(42.1)	
	Civil servant	13(12.2)	48(22.4)	
	Small business	21(19.6)	40(18.7)	
	Others	10(9.3)	20(9.3)	
Family size	3 to 5	7(6.5)	159(74.3)	X^2^ = 134.74, *P* < 0.001
	6 and above	100(93.5)	55(25.7)	
Family income	Below 5000 ETB	53(49.5)	82(38.3)	X^2^ = 1.93, *P* > 0.05
	6000 to 10, 000 ETB	46(43.0)	119(55.6)	
	Above 10, 000 ETB	8(7.5)	13(6.1)	

Among all participants, 67.3% and 15.0% of the women had a birth interval less than two years between consecutive births among cases and controls respectively. Additionally, 64.5% of the women did not attend ANC among the case group, and 11.7% among the control group. In addition to these, 20.6% of the women in the case group and 55.6% in the control group were had health center ANC follow-up (Table 2).

**Table 2:** Reproductive health-related characteristics in the last 9 months of preterm birth among mothers who gave birth at public hospitals in Bahir dar, Amhara regional state, and Northwest Ethiopia 2024 (*n* = 107 cases and 214 controls)

Predictor variables	Variable category	Cases	Controls	Statistics (X^2^), *P*-value
		N (%)	N (%)	
Birth interval	>=3 years	13(12.1)	125(58.4)	X^2^ = 100.1, *P* < 0.001
	2 to 3 years	22(20.6)	57(26.6)	
	< 2 years	72(67.3)	32(15.0)	
Attending ANC	Yes	38(35.5)	189(88.3)	X^2^ = 94.66, *P* < 0.001
	No	69(64.5)	25(11.7)	
Place of ANC	HC	22(20.6)	119(55.6)	X^2^ = 86.54, *P* < 0.001
	Hospital	15(14.0)	71(33.2)	
	No ANC visit	70(65.4)	24(11.2)	

Among all participants, 23.3% of the cases experienced danger signs during pregnancy and obstetrics complications such as vaginal bleeding, premature rupture of membranes (PROM), and a history of still birth. Similarly, 21.5% of the controls experienced danger signs during pregnancy and obstetrics complications. Among the cases, 39.1% had chronic hypertension followed by anemia (24.3%). In comparison, 3.3% and 7.9% of the controls had chronic hypertension and anemia, respectively (Table 3).

**Table 3:** Obstetrics complication related characteristics in the last 9 months of mothers who gave birth at public hospitals in Bahir dar, Amhara regional state, and Northwest Ethiopia 2024 (*n* = 107 cases and 214 controls)

Predictor variables	Variable category	Cases	Controls	Statistics (X^2^), P-value
		N (%)	N (%)	
Danger sign of during pregnancy	Yes	25(23.3)	46(21.5)	X^2^ = 0.14, *P* > 0.05
	No	82(76.7)	168(78.5)	
Obstetrics complication	Vaginal bleeding	10(9.6)	18(8.5)	X^2^ = 0.10, *P* > 0.05
	PROM	7(6.5)	14(6.5)	
	History of still birth	8(7.4)	14(6.5)	
	No complications	82(76.8)	168(78.5)	
Types of complication	Malaria	9(8.4)	20(9.3)	X^2^ = 42.25, *P* < 0.001
	Anemia	26(24.3)	17(7.9)	
	Chronic HTN	42(39.1)	7(3.3)	
	Other complications	11(10.3)	34(15.8)	
	No complication	19(17.9)	136(63.7)	

Among all participants, 68.2% of the women in the case group and 60.7% in the control group reported alcohol use during pregnancy.

Additionally, 43.0% of the women among cases and 8.9% of the women among controls did not take iron supplements. Furthermore, among newborns, 52.3% of the cases and 5.6% of the controls had low birth weight ((LBW) defined as less than 2,500 g. Regarding maternal hypertension, 74.8% in the case group and 31.3% in the control group had a history of maternal hypertension (Table 4).

**Table 4:** Behavioral and nutritional factors of mothers in the last 12 months of mothers who gave birth at public hospitals in Bahir dar, Amhara regional state, and Northwest Ethiopia 2024 (*n* = 107 cases and 214 controls)

Predictor variables	Variable category	Cases	Controls	Statistics (X^2^), *P*-value
		N (%)	N (%)	
Alcohol use	Yes	73(68.2)	130(60.7)	X^2^ = 1.73, *P* > 0.05
	No	34(31.8)	84(39.3)	
Iron use	Yes	61(57.0)	195(91.1)	X^2^ = 48.96, *P* < 0.001
	No	46(43.0)	19(8.9)	
Duration of Iron use	Based on prescription	27(25.2)	86(40.2)	X^2^ = 32.69, *P* < 0.001
	By interruption	36(33.6)	111(51.9)	
	Not at all	44(41.2)	17(7.9)	
Gestational ages (weeks)	Below 37 weeks	107(100)	0(0)	We cannot calculate the X^2^ value since there are empty cells.
	37 and above weeks	0(0)	214(100)	
Birth weight	LBW(< 2,500 g)	56(52.3)	12(5.6)	X^2^ = 112.34, *P* < 0.001
	VLBW < 1,500 g	14(13.1)	6(2.8)	
	NBW (2,500-4,000 g)	37(34.6)	196(91.6)	
Maternal nutritional status	MAM (MUAC 18.5–21.0 cm)	18(16.8)	31(14.5)	X^2^ = 0.25, *P* > 0.05
	Normal (MUAC > = 21.0 cm)	89(83.2)	183(85.5)	
Maternal HTN	Yes	80(74.8)	67(31.3)	X^2^ = 55.82, *P* < 0.001
	No	27(25.2)	147(68.7)	
HIV status	Negative	92(86.0)	203(94.9)	X^2^ = 0.17, *P* > 0.05
	Unknown	15(14.0)	11(5.1)	

**Fig. 1:**
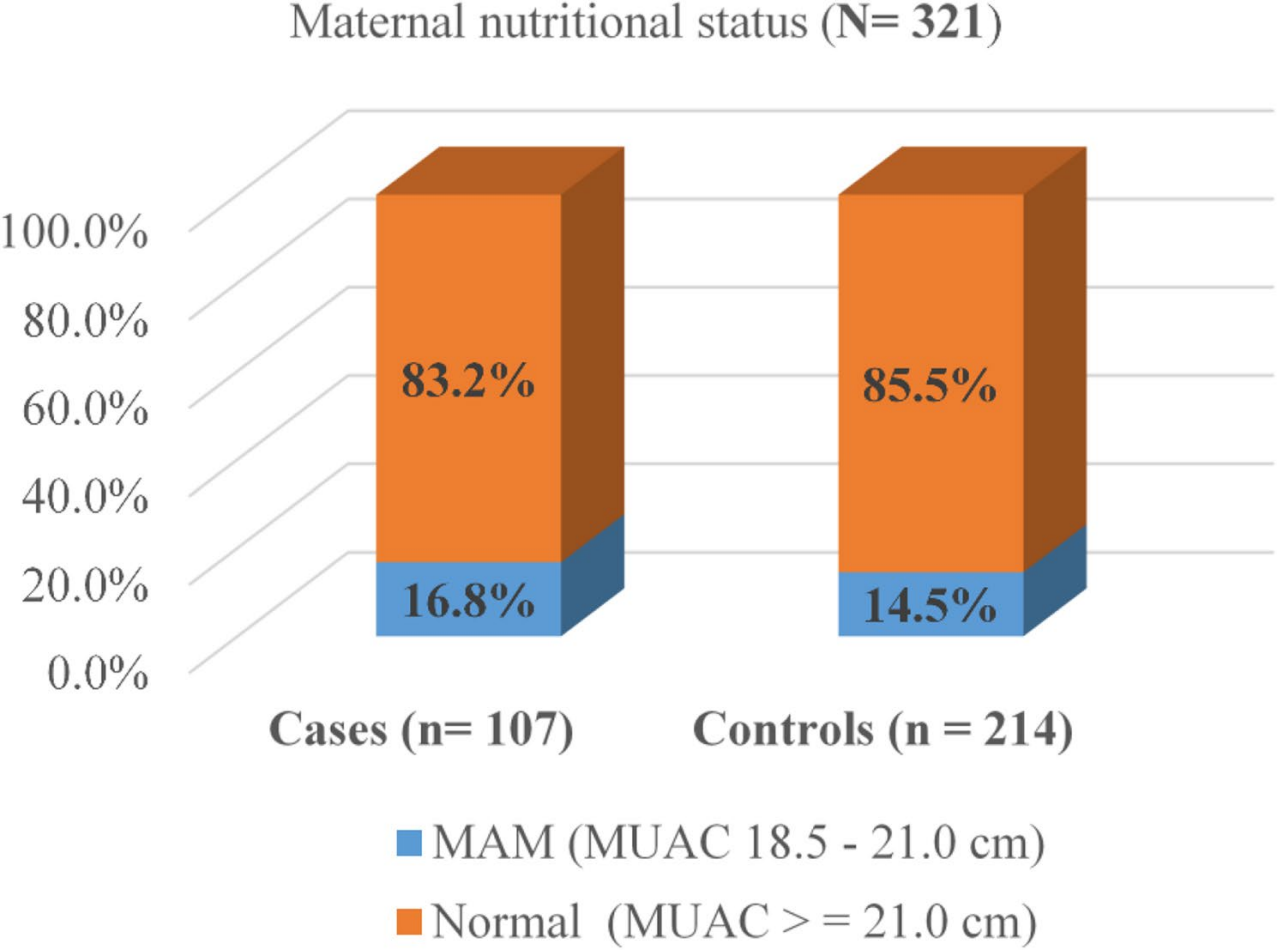
Nutritional status of women in the last 12 months among mothers who gave birth at public hospitals in Bahir Dar, Amhara Regional State, Northwest Ethiopia, 2024. Footnote: MAM: Moderate Acute Malnutrition; MUAC: Mid Upper Arm Circumference; cm: centimetres

The bar chart below illustrates the nutritional status of women, indicating that 16.8% of cases and 14.5% of controls had Moderate Acute Malnutrition (MAM) (Fig. 1).

**Fig. 2:**
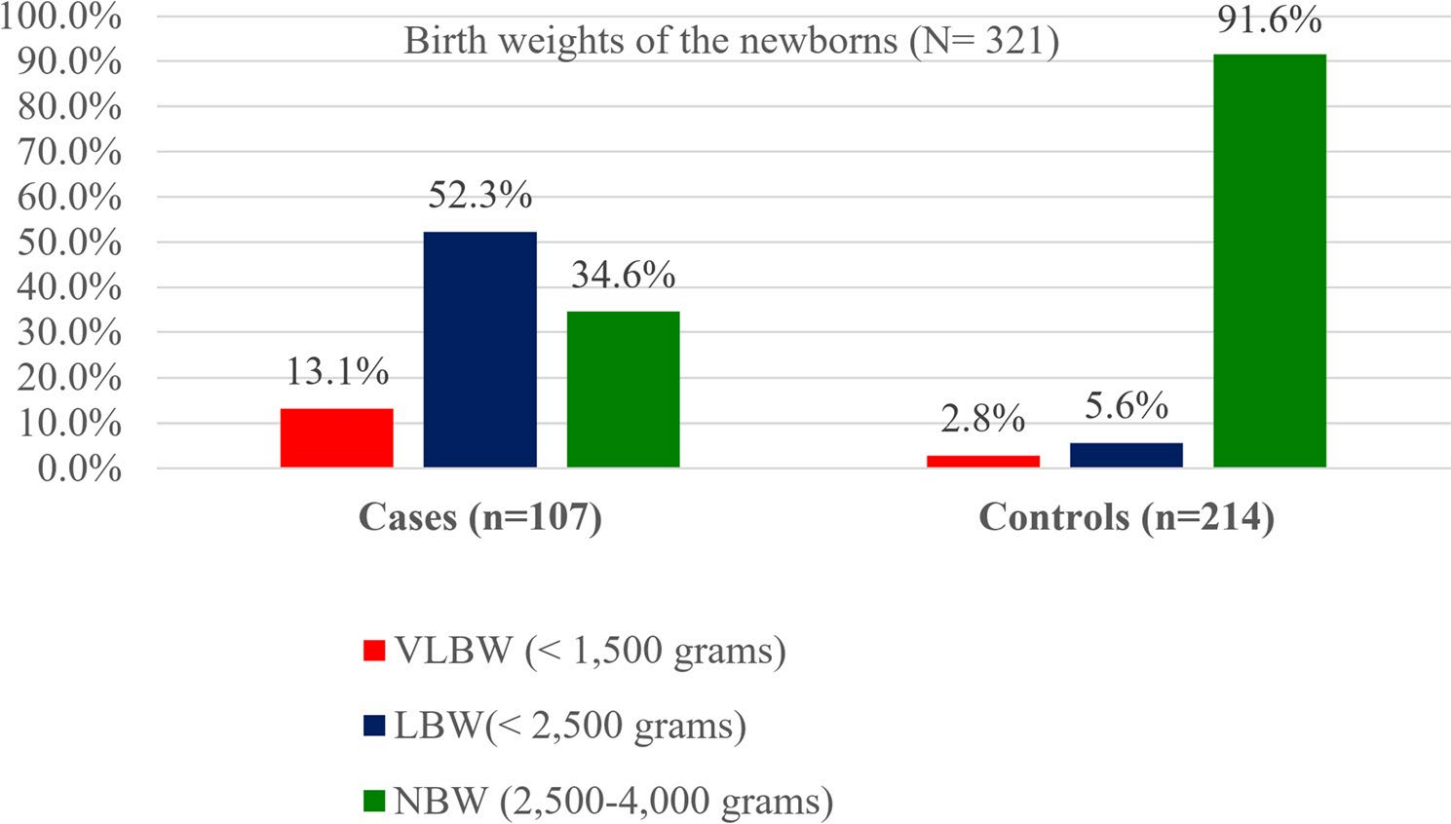
Patterns of newborn birth weight at public hospitals in Bahir Dar, Amhara Regional State, Northwest Ethiopia 2024. Footnote: NBW: Normal Birth Weight; LBW: Low Birth Weight; VLBW: Very Low Birth Weight

The bar chart below presents the birth weight distribution of newborns delivered at public hospitals in Bahir Dar (Fig. 2).

**Fig. 3:**
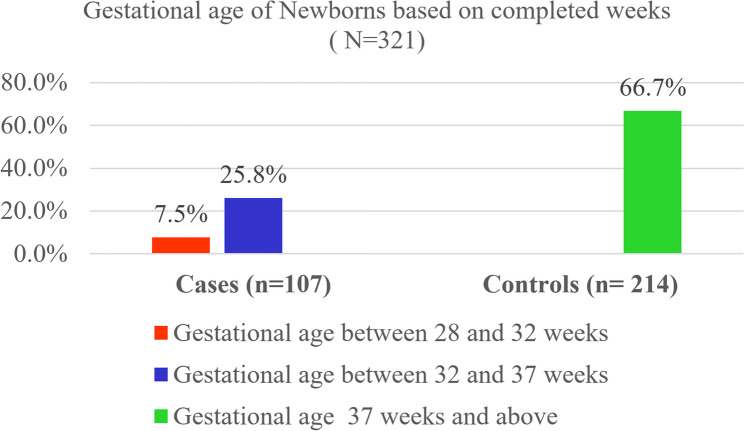
Gestational age of Newborns based on completed weeks at Public Hospitals in Bahir Dar, Amhara Regional State, Northwest Ethiopia 2024 (*n*= 107 cases and 214 controls)

The bar chart below illustrates the gestational age of newborns based on completed weeks. Among case group, 7.5% had a gestational age between 28 and 32 weeks, and 25.8% had a gestational age between 32 and 37 weeks. In contrast, 66.7% of the newborns in the control group had a gestational age of 37 weeks or above (Fig. 3).

### Determinant and interpretation of the determinant factors of preterm birth

In the final binary logistic regression model, five variables were found to be statistically significant factors of preterm: urban residence, family size of six or more, birth interval of less than two years, lack of antenatal care (ANC) follow-up, and not taking iron supplements during pregnancy.

The odds of mothers residing in urban areas were 79% less likely to experience preterm births compared to those living in rural areas (AOR: 0.21, 95% CI: 0.07, 0.60, *p*-value, 0.003).

The odds of women with a family size of six or more were 29 times more likely to experience preterm birth compared to those with a family size of three or fewer (AOR: 29, 95% CI: 6.29, 140.51, *p*-value, **<** 0 0.001).

The odds of women with a birth interval of less than two years between consecutive births were 17 times more likely to experience preterm birth compared to those with a birth interval of three years or more (AOR: 17, 95% CI: 4.77, 65.91, *p*-value, **<** 0.001).

The odds of women who had no antenatal care (ANC) follow-up were 5 times more likely to experience preterm birth compared to those who received ANC follow-up (AOR: 5, 95% CI: 1.68, 14.77, *p*-value, 0.004).

The odds of women who did not take iron with folic acid during pregnancy were 5 times more likely to experience preterm birth compared to those who did (AOR: 5, 95% CI: 1.69, 18.29, *p*-value, 0.005) by keeping other factors constant for all interpretations (Table 5).

**Table 5:** The bi-variable and multi-variable logistic regression analysis of preterm birth among mothers who gave birth at public hospitals in Bahir dar, Amhara regional state, Northwest Ethiopia 2024 (*n* = 107 cases and 214 controls). Where: DM; diabetics milieus, HTN; hypertension, UTI; urinary tract infection

Variables	Variable category	Preterm birth		COR [95%CI]	AOR[95%CI]	*P*-value
		Yes	No			
Residence	Rural	85	63	1	1	
	Urban	22	151	0.1 (0.06, 0.18) *	**0.21(0.07**,** 0.60)**	0.003**
Family size	Below 3	4	74	1	1	
	3 to 5	3	85	0.6(0.14, 3.01)	1.1(0.15,7.97) *	0.921
	6 and above	100	55	33.6(11.64,96.94) *	**29(6.29**,** 140.51)**	< 0 0.001**
Birth interval	>=3 years	13	125	1	1	
	2 to 3 years	22	57	3. 71(1.74, 7.88) *	6.93(0.91, 11.14)	0.068
	< 2 years	72	32	21.6(10.67, 43. 86) *	**17(4.77**,** 65.91)**	< 0.001**
Attending ANC	Yes	38	189	1	1	
	No	69	25	13.7(7.72, 24.39)*	**5(1.68**,** 14.77)**	0.004**
Types of medical complication	No complication	19	136	1	1	
	Malaria	9	20	1.1(0.35, 3.74)	0.21(0.02, 1. 74)	0.149
	Anemia	26	17	3.9(1.35, 11. 41)*	1.76(0.27, 11. 48)	0.554
	STI	3	2	3. 8(0.52, 28.24)	5 (0.23, 127.0)	0.542
	Chronic HTN	42	5	21.6(6.04, 77.18)*	4 (0.55, 32.65)	0.163
	DM	1	16	0.16(0.01, 1. 45)	0.08 (0.01, 1.42)	0.087
	UTI	7	18	0.35(0.13, 0.97)*	0.3 (0.05, 1.71)	0.183
Alcohol use	Yes	73	130	1	1	
	No	34	84	0.72(0.44, 1.17)	0.4(0.15, 1.52)	0.215
Iron use	Yes	61	195	1	1	
	No	46	19	7.7(4.21, 14.20)*	**5(1.69**,** 18.29)**	0.005**

1_Reference category

*Associated variables based on bi-variable selection criteria

**Statistically significant at multi-variable analysis with 5% level of significance, CI- confidence interval

## Discussion

In our study, the odds of mothers residing in urban areas were 79% less likely to experience preterm births compared to those living in rural areas (AOR: 0.21, 95% CI: 0.07, 0.60).

This finding was supported by studies in Ethiopian showed that rural mothers face delays in seeking, accessing, and receiving care which resulted pregnancy complications of preterm birth [29, 30]. In a similar manner, sub-Saharan African studies correlate rural residence with poor prenatal care and increased risk through reduced access to healthcare and quality ADDIN EN.CITE [13]. The urban-rural dichotomy is not exclusive to Africa but also in Asian nations such as India and Bangladesh, preterm birth among rural mothers is greater due to inadequate healthcare infrastructure and reduced ANC visits ADDIN EN.CITE [31, 32]. A Nigerian study also ascribes rural socio-economic constraints and substandard maternal care to increased adverse outcomes, mirroring South Asian trends ADDIN EN.CITE [31].

Even among high-income nations like the US, Canada, and Australia, international rates reflect preterm birth increased among rural women because of isolation and late access to neonatal care ADDIN EN.CITE [33–35]. These consistent findings underscore that, despite urban healthcare advancements, rural populations worldwide face persistent challenges, necessitating targeted improvements in maternal health infrastructure and access.

The odds of women with a family size of six or more were 29 times more likely to experience preterm birth compared to those with a family size of three or fewer (AOR: 29, 95% CI: 6.29, 140.51). This is consistent with previous research that has repeatedly demonstrated that larger family sizes are related to an increased risk of adverse birth outcomes such as preterm birth [36].

Research in Ethiopia, sub-Saharan Africa, and another in the US indicates that higher family sizes result from cultural and socioeconomic determinants ADDIN EN.CITE [37–39]. Yet the larger the family size, the thinner the family resources become and, as such, may no longer be enough to provide appropriate maternal nutrition as well as increased exposure to lower access to identify risk determinants for preterm birth. For instance, research in Ethiopia found that women from larger families were the least likely to receive antenatal care, a pattern linked to poorer birth outcomes [40, 41]. These observations suggest that higher parity may heighten the risk of preterm birth, possibly due to a combination of factors such as maternal health, prior pregnancies, and biological or environmental conditions.

The odds of women with a birth interval of less than two years between consecutive births were 17 times more likely to experience preterm birth compared to those with a birth interval of three years or more (AOR: 17, 95% CI: 4.77, 65.91). Align a prior study in Ethiopia highlighting the risks of short inter-pregnancy intervals [37]. In Ethiopia, short birth intervals are prevalent, with approximately 53% of women having intervals under 24 months [37]. Such high prevalence is disconcerting because short inter-pregnancy intervals are linked to poor perinatal outcomes, such as preterm birth [42]. A 25.9% preterm birth rate was, for instance, reported in an Ethiopian study in mothers with short intervals, while that of their counterparts with optimal intervals was at 2.9% [37].

Similarly, in sub-Saharan Africa, 57% of births were within intervals less than 24 months, with the result of high preterm birth rates within the region. Key contributing factors include limited access to family planning, cultural practices, and socioeconomic challenges, all of which must be addressed to enhance maternal and neonatal health outcomes in the region [41, 43].

Globally, a well-established connection exists between short inter-pregnancy intervals and adverse birth outcomes, such as preterm birth. A systematic review found that intervals of less than six months heighten the risks of preterm birth, small-for-gestational-age infants, and infant mortality [29, 44]. These findings emphasize the critical need to follow recommended birth spacing guidelines to minimize the risks of adverse perinatal outcomes [29, 44].

The odds of women who had no antenatal care (ANC) follow-up were 5 times more likely to experience preterm birth compared to those who received ANC follow-up (AOR: 5, 95% CI: 1.68, 14.77). This highlighting the critical importance of prenatal care in averting preterm births [45]. Preterm birth contributes about 10.48% of total births in Ethiopia, a rate that is fueled by the lack or poor quality of ANC services in some parts of the country [46]. A study in Ethiopia demonstrated that the absence of sufficient ANC increases preterm birth risk by limiting opportunities for early risk detection and preventive interventions [32].

In sub-Saharan Africa, the lack of ANC services is a primary driver of adverse pregnancy outcomes, such as preterm birth [46]. Enhanced ANC provision could alleviate the burden of preterm births in these areas. Research has shown that ANC visits are linked to reduced preterm birth rates, reinforcing the importance of accessible ANC services in regions with elevated preterm birth prevalence [47].

Globally, the absence of ANC is a well-established risk factor for preterm birth. According to the World Health Organization, prematurity remains the leading cause of death among children under five, with significant survival disparities worldwide particularly it is high in low-income settings [48].

The odds of women who did not take iron with folic acid during pregnancy were 5 times more likely to experience preterm birth compared to those who did (AOR: **5**, 95% CI: 1.69, 18.29).

It deserves further study in the context of the highly complex relationship between micronutrient supplementation, pregnancy outcomes, and maternal health. Iron deficiency anemia is one of the great public health concerns across the whole of sub-Saharan Africa, and thus supplementation programs are widely implemented for its prevention. A study from Ethiopia found that nearly 35% of pregnant women were affected by anemia, therefore becoming one major cause of preterm birth and a bad pregnancy outcome [49, 50]. Evidence underlines that poorly monitored iron supplementation may cause oxidative stress and possibly relate it to preterm birth in some populations [51, 52]. The effects of such supplementation, though, are sometimes quite unexpected. Evidence from elsewhere in sub-Saharan Africa indicates that although iron supplementation is a requirement to prevent anemia and to promote maternal health, high iron intake may increase the risk of infections and might also contribute to pregnancy complications such as preterm birth [47]. One study from Uganda has reported an increased risk of preterm birth for women receiving high doses of iron supplementation compared with those receiving lower doses or no supplementation [53].

Globally, supplementation of iron is generally recommended for anemia prevention, but the evidence regarding its relationship with preterm birth is mixed. A systematic review and meta-analysis from high-income countries found no significant effect on the risk of preterm birth [54]. On the other hand, some studies indicate that iron supplements, particularly when given in high doses, might increase oxidative stress, potentially harmful to pregnancy outcomes [55]. Other studies point out that timing and dosing of iron supplements are major determinants of whether they will contribute to maternal health and pregnancy outcomes [56].

Conclusion

In conclusion, this study identified several significant determinants of preterm birth. Mothers residing in urban areas had significantly lower odds of experiencing preterm birth compared to their rural counterparts. Conversely, having a larger family size, shorter birth intervals, lack of antenatal care follow-up, and not taking iron with folic acid supplements during pregnancy were associated with increased odds of preterm birth. These findings highlight that urban residence is associated with a reduced risk of preterm birth. Additionally, having a small family size, maintaining adequate birth spacing, taking iron with folic acid supplements, and attending antenatal care follow-ups were also protective factors against preterm birth.

## Recommendation

Based on the findings, we recommend strengthening maternal health services, particularly among women in rural areas, to reduce the risk of preterm birth. Interventions should focus on promoting adequate birth spacing, increasing access to and utilization of antenatal care, and ensuring the availability and intake of iron with folic acid supplements during pregnancy.

Public health programs should also address the needs of women with larger family size by providing targeted education and support to mitigate associated risks.

### Strengths of the study

1. Appropriate Study Design: The case-control design is well-suited for exploring risk factors of relatively rare outcomes like preterm birth.
2. Primary Data Collection: Data were collected through direct interviews, which helped to ensure completeness and consistency, and allowed clarification of unclear responses.
3. Use of Multivariable Analysis: Adjusting for potential confounders using logistic regression enhanced the validity of the associations found.
4. Local Relevance: The study provides context-specific evidence from public referral hospitals in Bahir Dar city, which can guide local public health interventions.
5. Efficient Resource Use: The case-control approach allowed the investigation of multiple risk factors without requiring a large cohort, making it cost-effective and time-efficient.

### Limitations of the study

Despite direct interviews, participants may still have had difficulty accurately recalling past exposures such as supplement intake or timing of previous births. Participants may have over reported desirable behaviors (e.g., ANC visits or supplement intake).

## Data Availability

All the relevant information was included in the manuscript.

## Abbreviations

AIDS: Acquired immune deficiency Syndrome
ANC: Ante Natal Care
ANRS: Amhara National Regional State
APH: Ante Par tum Hemorrhage
CEO: Chief Executive Officer
CI: Confidence Interval
CUFY: Children Under Five Years
DM: Diabetes Militus
EDHS: Ethiopia Demographic and Health Survey
ETB: Ethiopian Birr
FHRH: Felege Hiowt Referral Hospital
GA: Gestational Age
HGB: Haemoglobin
HIV: Human Immune Virus
IDA: Iron Deficiency Anemia
LMIC: Low-and Middle-Income Countries
LNMP: Last Normal Menstrual Period
MUAC: Mid Upper Arm Circumference
MAM: Moderate Acute Malnutrition
NM: Newborn Mortality
OR: Odds Ratio
PIH: Pregnancy Induced Hypertension
PROM: Premature Rupture of Membrane
PTB: Preterm Birth
SAM: Sever Acute Malnutrition
SDG: Sever Acute Malnutrition
SPSS: Social and Psychological Science Statistical Software
STI: Sexual Transmitted Diseases
TGSRH: Tibebe Ghion Specialized Referal Hospital
U/S: Ultra Sound
U5MR: Under 5 Mortality Rate
UFM: Under-Five Mortality
UNSDG: United Nations Sustainable Development Goals
UTI: Urinary Tract Infections
WHO: World Health Organization
